# {2,2′-[1,1′-(Ethyl­enedioxy­dinitrilo)diethyl­idyne]di-1-naphtholato}copper(II)

**DOI:** 10.1107/S1600536809023010

**Published:** 2009-06-20

**Authors:** Wen-Kui Dong, Jian-Chao Wu, Jian Yao, Shang-Sheng Gong, Jun-Feng Tong

**Affiliations:** a School of Chemical and Biological Engineering, Lanzhou Jiaotong University, Lanzhou 730070, People’s Republic of China

## Abstract

The title complex, [Cu(C_26_H_22_N_2_O_4_)],  is isostructural with its Ni analogue. All intramolecular distances and angles are very similar for the two structures, whereas the packing of the molecules, including C—H⋯O and C—H⋯π interactions, are slightly different.

## Related literature

For transition metal complexes with multidentate salen-type ligands, see: Akine *et al.* (2005 ▶); Dong *et al.* (2009*a* ▶,*b* ▶); Katsuki (1995 ▶); Ray *et al.* (2003 ▶); Sun *et al.* (2008 ▶). For the isostructural Ni complex, see: Dong *et al.* (2009*c* ▶).
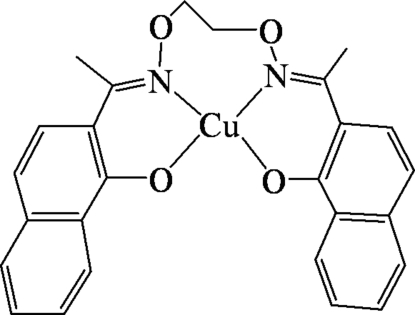


## Experimental

### 

#### Crystal data

[Cu(C_26_H_22_N_2_O_4_)]
*M* *_r_* = 490.00
Monoclinic, 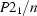

*a* = 13.0288 (17) Å
*b* = 7.8934 (12) Å
*c* = 21.292 (2) Å
β = 103.217 (2)°
*V* = 2131.7 (5) Å^3^
*Z* = 4
Mo *K*α radiation
μ = 1.06 mm^−1^
*T* = 298 K
0.41 × 0.17 × 0.07 mm

#### Data collection

Bruker SMART 1000 CCD diffractometer
Absorption correction: multi-scan (*SADABS*; Sheldrick, 1996 ▶) *T*_min_ = 0.670, *T*_max_ = 0.929
10698 measured reflections
3753 independent reflections
2278 reflections with *I* > 2σ(*I*)
*R*_int_ = 0.051

#### Refinement

*R*[*F* ^2^ > 2σ(*F* ^2^)] = 0.043
*wR*(*F* ^2^) = 0.108
*S* = 1.03
3753 reflections
298 parameters
H-atom parameters constrained
Δρ_max_ = 0.29 e Å^−3^
Δρ_min_ = −0.41 e Å^−3^

### 

Data collection: *SMART* (Siemens, 1996 ▶); cell refinement: *SAINT* (Siemens, 1996 ▶); data reduction: *SAINT*; program(s) used to solve structure: *SHELXS97* (Sheldrick, 2008 ▶); program(s) used to refine structure: *SHELXL97* (Sheldrick, 2008 ▶); molecular graphics: *SHELXTL* (Sheldrick, 2008 ▶); software used to prepare material for publication: *SHELXTL*.

## Supplementary Material

Crystal structure: contains datablocks global, I. DOI: 10.1107/S1600536809023010/at2815sup1.cif

Structure factors: contains datablocks I. DOI: 10.1107/S1600536809023010/at2815Isup2.hkl

Additional supplementary materials:  crystallographic information; 3D view; checkCIF report

## Figures and Tables

**Table 1:** Hydrogen-bond geometry (Å, °)

*D*—H⋯*A*	*D*—H	H⋯*A*	*D*⋯*A*	*D*—H⋯*A*
C16—H16*A*⋯O3^i^	0.96	2.64	3.375 (5)	134
C23—H23⋯O2^ii^	0.93	2.43	3.261 (5)	149
C4—H4*C*⋯*Cg*8^i^	0.96	2.68	3.564 (6)	153

Symmetry codes: (i) 
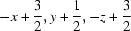
; (ii) 
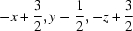
. *Cg*8 is the centroid of the C21–C26 ring.

**Fig. 1.**
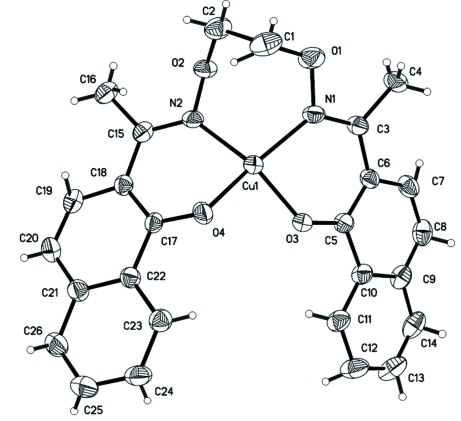
The molecule structure of the title complex possessing a crystallographic twofold screw axis passing through the middle point of (–O)–H2C—CH2–(O–) unit (symmetry code: 1/2 - x, 1/2 + y, 1/2 - z). Displacement ellipsoids for non-hydrogen atoms are drawn at the 30% probability level.

## supplementary crystallographic information

### Comment

Transition metal complexes with multidentate salen-type ligands are very interesting in modern coordination chemistry because they have mono-, di- or tri-nuclear metal complexes with important stereochemistry (Katsuki *et al.*, 1995; Akine *et al.*, 2005; Dong *et al.*, 2009*b*). Metal derivatives of salen-type compounds have been investigated extensively, and copper(II) complexes play a major role in both synthetic and structural research (Ray *et al.*, 2003; Dong *et al.*, 2009*a*).

In this paper, a new mononuclear copper(II) complex with salen-type bisoxime chelating ligand, 2,2'-[1,1'-ethylenedioxybis(nitriloethylidyne)]dinaphthol, has been synthesized (Sun *et al.*, 2008). The X-ray crystallography of the title complex (Fig. 1) reveals the complex crystallizes in the monoclinic system, with P2_1_/c space group. There is a crystallographic twofold screw axis (symmetry code: 1/2 - *x*, 1/2 + *y*, 1/2 - *z*). The dihedral angle between the coordination plane of O3—Cu1—N1 and that of O4—Cu1—N2 is 26.53°, indicating slight distortion toward tetrahedral geometry from the square planar structure [Cu1—O3: 1.876 (3) Å; Cu1—O4: 1.895 (3) Å; Cu1—N1: 1.976 (3) Å; Cu1—N2: 1.947 (3) Å]), with a mean deviation of 0.016 Å from the N_2_O_2_ plane. The crystal structure is further stabilized by intermolecular C16—H16A···O3, C23—H23···O2 hydrogen bonds and C4—H4C···π interactions (Table 1), which link neighbouring molecules into extended chains along the *c* axis.

### Experimental

A solution of Cu(II) acetate monohydrate (1.7 mg, 0.0085 mmol) in ethanol (5 ml) was added dropwise to a solution of 2,2'-[1,1'-ethylenedioxybis(nitriloethylidyne)]dinaphthol (3.4 mg, 0.0079 mmol) in dichloromethane (5 ml). The colour of the mixing solution turns to brown, immediately, and was allowed to stand at room temperature for about one week, the solvent was partially evaporated and obtained dark-brown needle-like single crystals suitable for X-ray crystallographic analysis.

### Refinement

H atoms were treated as riding atoms with distances C—H = 0.96 (CH_3_), C—H = 0.97 (CH_2_), or 0.93 Å (CH), and *U*_iso_(H) = 1.2 *U*_eq_(C) and 1.5 *U*_eq_(C_methyl_).

### Crystal data

**Table:**

[Cu(C_26_H_22_N_2_O_4_)]	*F*(000) = 1012
*M**_r_* = 490.00	*D*_x_ = 1.527 Mg m^−^^3^
Monoclinic, *P*2_1_/*n*	Mo *K*α radiation, λ = 0.71073 Å
Hall symbol: -P 2yn	Cell parameters from 2535 reflections
*a* = 13.0288 (17) Å	θ = 3.0–25.3°
*b* = 7.8934 (12) Å	µ = 1.06 mm^−^^1^
*c* = 21.292 (2) Å	*T* = 298 K
β = 103.217 (2)°	Needle, dark-brown
*V* = 2131.7 (5) Å^3^	0.41 × 0.17 × 0.07 mm
*Z* = 4	

### Data collection

**Table:**

Bruker SMART 1000 CCD area-detector diffractometer	3753 independent reflections
Radiation source: fine-focus sealed tube	2278 reflections with *I* > 2σ(*I*)
graphite	*R*_int_ = 0.051
φ and ω scans	θ_max_ = 25.0°, θ_min_ = 1.7°
Absorption correction: multi-scan (*SADABS*; Sheldrick, 1996)	*h* = −15→13
*T*_min_ = 0.670, *T*_max_ = 0.929	*k* = −9→9
10698 measured reflections	*l* = −25→23

### Refinement

**Table:**

Refinement on *F*^2^	Primary atom site location: structure-invariant direct methods
Least-squares matrix: full	Secondary atom site location: difference Fourier map
*R*[*F*^2^ > 2σ(*F*^2^)] = 0.043	Hydrogen site location: inferred from neighbouring sites
*wR*(*F*^2^) = 0.108	H-atom parameters constrained
*S* = 1.03	*w* = 1/[σ^2^(*F*_o_^2^) + (0.0328*P*)^2^ + 2.0905*P*] where *P* = (*F*_o_^2^ + 2*F*_c_^2^)/3
3753 reflections	(Δ/σ)_max_ = 0.001
298 parameters	Δρ_max_ = 0.29 e Å^−^^3^
0 restraints	Δρ_min_ = −0.41 e Å^−^^3^

### Special details

**Table:**

Geometry. All e.s.d.'s (except the e.s.d. in the dihedral angle between two l.s. planes) are estimated using the full covariance matrix. The cell e.s.d.'s are taken into account individually in the estimation of e.s.d.'s in distances, angles and torsion angles; correlations between e.s.d.'s in cell parameters are only used when they are defined by crystal symmetry. An approximate (isotropic) treatment of cell e.s.d.'s is used for estimating e.s.d.'s involving l.s. planes.
Refinement. Refinement of *F*^2^ against ALL reflections. The weighted *R*-factor *wR* and goodness of fit *S* are based on *F*^2^, conventional *R*-factors *R* are based on *F*, with *F* set to zero for negative *F*^2^. The threshold expression of *F*^2^ > σ(*F*^2^) is used only for calculating *R*-factors(gt) *etc*. and is not relevant to the choice of reflections for refinement. *R*-factors based on *F*^2^ are statistically about twice as large as those based on *F*, and *R*- factors based on ALL data will be even larger.

### Fractional atomic coordinates and isotropic or equivalent isotropic displacement parameters (Å^2^)

**Table:**

	*x*	*y*	*z*	*U*_iso_*/*U*_eq_	
Cu1	0.73155 (4)	0.18038 (7)	0.77719 (2)	0.04608 (19)	
N1	0.7004 (3)	0.1798 (4)	0.86373 (14)	0.0465 (9)	
N2	0.8811 (2)	0.2362 (4)	0.79867 (15)	0.0452 (9)	
O1	0.7720 (2)	0.1113 (4)	0.91807 (14)	0.0680 (9)	
O2	0.9266 (2)	0.2769 (4)	0.86424 (13)	0.0512 (8)	
O3	0.5864 (2)	0.1927 (4)	0.74053 (12)	0.0555 (8)	
O4	0.7494 (2)	0.0995 (4)	0.69654 (13)	0.0562 (8)	
C1	0.8537 (4)	0.0179 (6)	0.9005 (2)	0.0689 (14)	
H1A	0.8289	−0.0266	0.8572	0.083*	
H1B	0.8730	−0.0772	0.9297	0.083*	
C2	0.9486 (3)	0.1269 (6)	0.9028 (2)	0.0637 (13)	
H2A	0.9779	0.1594	0.9472	0.076*	
H2B	1.0015	0.0610	0.8882	0.076*	
C3	0.6172 (3)	0.2413 (5)	0.87978 (19)	0.0465 (11)	
C4	0.6114 (4)	0.2381 (7)	0.94956 (19)	0.0661 (14)	
H4A	0.6453	0.1376	0.9697	0.099*	
H4B	0.5389	0.2384	0.9523	0.099*	
H4C	0.6463	0.3362	0.9711	0.099*	
C5	0.5175 (3)	0.2722 (5)	0.76545 (19)	0.0435 (10)	
C6	0.5294 (3)	0.3069 (5)	0.83128 (19)	0.0447 (10)	
C7	0.4498 (4)	0.4048 (6)	0.8504 (2)	0.0572 (12)	
H7	0.4581	0.4316	0.8938	0.069*	
C8	0.3623 (4)	0.4603 (6)	0.8078 (2)	0.0622 (13)	
H8	0.3129	0.5248	0.8225	0.075*	
C9	0.3450 (3)	0.4223 (6)	0.7418 (2)	0.0532 (12)	
C10	0.4212 (3)	0.3260 (5)	0.7203 (2)	0.0466 (10)	
C11	0.4048 (3)	0.2850 (6)	0.6547 (2)	0.0553 (12)	
H11	0.4546	0.2203	0.6404	0.066*	
C12	0.3158 (4)	0.3395 (7)	0.6113 (2)	0.0737 (15)	
H12	0.3054	0.3109	0.5679	0.088*	
C13	0.2418 (4)	0.4369 (7)	0.6322 (3)	0.0781 (16)	
H13	0.1819	0.4739	0.6027	0.094*	
C14	0.2557 (4)	0.4787 (6)	0.6952 (3)	0.0694 (14)	
H14	0.2057	0.5458	0.7082	0.083*	
C15	0.9430 (3)	0.2702 (5)	0.7603 (2)	0.0441 (10)	
C16	1.0539 (3)	0.3300 (6)	0.7877 (2)	0.0596 (12)	
H16A	1.0532	0.4490	0.7969	0.089*	
H16B	1.0961	0.3103	0.7569	0.089*	
H16C	1.0831	0.2690	0.8266	0.089*	
C17	0.8113 (3)	0.1659 (5)	0.66393 (18)	0.0388 (10)	
C18	0.9056 (3)	0.2506 (5)	0.69117 (19)	0.0418 (10)	
C19	0.9665 (3)	0.3161 (6)	0.6492 (2)	0.0530 (11)	
H19	1.0285	0.3736	0.6672	0.064*	
C20	0.9390 (3)	0.2994 (6)	0.5845 (2)	0.0564 (12)	
H20	0.9819	0.3440	0.5592	0.068*	
C21	0.8443 (3)	0.2135 (5)	0.5550 (2)	0.0485 (11)	
C22	0.7806 (3)	0.1459 (5)	0.59432 (19)	0.0427 (10)	
C23	0.6864 (3)	0.0651 (5)	0.5649 (2)	0.0502 (11)	
H23	0.6435	0.0213	0.5904	0.060*	
C24	0.6560 (4)	0.0493 (6)	0.4991 (2)	0.0613 (13)	
H24	0.5929	−0.0043	0.4802	0.074*	
C25	0.7202 (4)	0.1138 (7)	0.4606 (2)	0.0712 (15)	
H25	0.7005	0.1010	0.4160	0.085*	
C26	0.8112 (4)	0.1952 (7)	0.4879 (2)	0.0641 (13)	
H26	0.8524	0.2398	0.4615	0.077*	

### Atomic displacement parameters (Å^2^)

**Table:**

	*U*^11^	*U*^22^	*U*^33^	*U*^12^	*U*^13^	*U*^23^
Cu1	0.0401 (3)	0.0603 (4)	0.0372 (3)	−0.0043 (3)	0.0077 (2)	−0.0032 (3)
N1	0.046 (2)	0.056 (2)	0.0345 (19)	−0.0113 (19)	0.0022 (16)	0.0057 (18)
N2	0.043 (2)	0.050 (2)	0.039 (2)	−0.0019 (17)	0.0021 (16)	−0.0126 (17)
O1	0.060 (2)	0.093 (3)	0.0447 (19)	−0.0031 (19)	−0.0005 (16)	0.0194 (18)
O2	0.0535 (18)	0.051 (2)	0.0445 (17)	−0.0028 (15)	0.0010 (13)	−0.0102 (15)
O3	0.0415 (16)	0.087 (2)	0.0368 (16)	−0.0011 (17)	0.0062 (13)	−0.0113 (16)
O4	0.0506 (18)	0.077 (2)	0.0449 (17)	−0.0232 (16)	0.0196 (14)	−0.0171 (16)
C1	0.068 (3)	0.055 (3)	0.070 (3)	0.002 (3)	−0.012 (3)	0.018 (3)
C2	0.056 (3)	0.064 (3)	0.062 (3)	0.013 (3)	−0.005 (2)	0.002 (3)
C3	0.052 (3)	0.051 (3)	0.037 (2)	−0.019 (2)	0.011 (2)	0.000 (2)
C4	0.068 (3)	0.093 (4)	0.039 (3)	−0.018 (3)	0.015 (2)	0.002 (3)
C5	0.041 (2)	0.046 (3)	0.044 (2)	−0.015 (2)	0.011 (2)	−0.001 (2)
C6	0.047 (2)	0.048 (3)	0.041 (2)	−0.010 (2)	0.015 (2)	−0.005 (2)
C7	0.066 (3)	0.060 (3)	0.051 (3)	−0.016 (3)	0.025 (3)	−0.005 (2)
C8	0.058 (3)	0.051 (3)	0.085 (4)	−0.007 (3)	0.032 (3)	−0.006 (3)
C9	0.045 (3)	0.043 (3)	0.073 (3)	−0.011 (2)	0.017 (2)	0.007 (3)
C10	0.041 (2)	0.047 (3)	0.051 (3)	−0.013 (2)	0.008 (2)	0.004 (2)
C11	0.044 (3)	0.060 (3)	0.059 (3)	−0.012 (2)	0.005 (2)	0.006 (2)
C12	0.058 (3)	0.090 (4)	0.063 (3)	−0.021 (3)	−0.008 (3)	0.018 (3)
C13	0.048 (3)	0.075 (4)	0.100 (5)	−0.006 (3)	−0.006 (3)	0.028 (4)
C14	0.045 (3)	0.057 (3)	0.106 (4)	−0.003 (2)	0.016 (3)	0.011 (3)
C15	0.039 (2)	0.037 (3)	0.056 (3)	0.0032 (19)	0.008 (2)	−0.009 (2)
C16	0.043 (3)	0.062 (3)	0.070 (3)	−0.004 (2)	0.006 (2)	−0.008 (3)
C17	0.033 (2)	0.040 (3)	0.045 (2)	0.0005 (19)	0.0113 (18)	−0.006 (2)
C18	0.039 (2)	0.042 (3)	0.045 (2)	0.002 (2)	0.0125 (19)	−0.003 (2)
C19	0.043 (2)	0.052 (3)	0.066 (3)	−0.004 (2)	0.017 (2)	−0.001 (3)
C20	0.057 (3)	0.062 (3)	0.057 (3)	−0.001 (3)	0.026 (2)	0.004 (3)
C21	0.051 (3)	0.051 (3)	0.046 (3)	0.009 (2)	0.013 (2)	0.006 (2)
C22	0.044 (2)	0.044 (3)	0.041 (2)	0.006 (2)	0.0108 (19)	−0.001 (2)
C23	0.049 (3)	0.050 (3)	0.050 (3)	0.001 (2)	0.010 (2)	−0.002 (2)
C24	0.055 (3)	0.077 (4)	0.045 (3)	0.004 (3)	−0.004 (2)	−0.008 (3)
C25	0.072 (4)	0.094 (4)	0.044 (3)	0.010 (3)	0.005 (3)	0.009 (3)
C26	0.066 (3)	0.078 (4)	0.051 (3)	0.008 (3)	0.019 (2)	0.016 (3)

### Geometric parameters (Å, °)

**Table:**

Cu1—O3	1.876 (3)	C10—C11	1.402 (6)
Cu1—O4	1.895 (3)	C11—C12	1.376 (6)
Cu1—N2	1.947 (3)	C11—H11	0.9300
Cu1—N1	1.976 (3)	C12—C13	1.385 (7)
N1—C3	1.302 (5)	C12—H12	0.9300
N1—O1	1.417 (4)	C13—C14	1.352 (7)
N2—C15	1.301 (5)	C13—H13	0.9300
N2—O2	1.423 (4)	C14—H14	0.9300
O1—C1	1.414 (5)	C15—C18	1.449 (5)
O2—C2	1.432 (5)	C15—C16	1.504 (5)
O3—C5	1.304 (5)	C16—H16A	0.9600
O4—C17	1.290 (4)	C16—H16B	0.9600
C1—C2	1.497 (6)	C16—H16C	0.9600
C1—H1A	0.9700	C17—C18	1.403 (5)
C1—H1B	0.9700	C17—C22	1.453 (5)
C2—H2A	0.9700	C18—C19	1.422 (5)
C2—H2B	0.9700	C19—C20	1.346 (6)
C3—C6	1.450 (6)	C19—H19	0.9300
C3—C4	1.505 (5)	C20—C21	1.421 (6)
C4—H4A	0.9600	C20—H20	0.9300
C4—H4B	0.9600	C21—C26	1.404 (6)
C4—H4C	0.9600	C21—C22	1.411 (5)
C5—C6	1.401 (5)	C22—C23	1.398 (5)
C5—C10	1.458 (5)	C23—C24	1.372 (5)
C6—C7	1.426 (6)	C23—H23	0.9300
C7—C8	1.357 (6)	C24—C25	1.394 (6)
C7—H7	0.9300	C24—H24	0.9300
C8—C9	1.402 (6)	C25—C26	1.357 (6)
C8—H8	0.9300	C25—H25	0.9300
C9—C10	1.407 (6)	C26—H26	0.9300
C9—C14	1.417 (6)		
			
O3—Cu1—O4	87.76 (11)	C11—C10—C5	120.1 (4)
O3—Cu1—N2	160.95 (14)	C9—C10—C5	120.5 (4)
O4—Cu1—N2	88.05 (12)	C12—C11—C10	120.7 (5)
O3—Cu1—N1	89.17 (12)	C12—C11—H11	119.7
O4—Cu1—N1	159.73 (14)	C10—C11—H11	119.7
N2—Cu1—N1	100.93 (13)	C11—C12—C13	120.0 (5)
C3—N1—O1	111.1 (3)	C11—C12—H12	120.0
C3—N1—Cu1	127.2 (3)	C13—C12—H12	120.0
O1—N1—Cu1	121.7 (3)	C14—C13—C12	120.6 (5)
C15—N2—O2	113.0 (3)	C14—C13—H13	119.7
C15—N2—Cu1	129.1 (3)	C12—C13—H13	119.7
O2—N2—Cu1	116.9 (2)	C13—C14—C9	121.4 (5)
C1—O1—N1	112.2 (3)	C13—C14—H14	119.3
N2—O2—C2	111.0 (3)	C9—C14—H14	119.3
C5—O3—Cu1	125.3 (2)	N2—C15—C18	120.2 (4)
C17—O4—Cu1	124.9 (3)	N2—C15—C16	120.0 (4)
O1—C1—C2	110.9 (4)	C18—C15—C16	119.8 (4)
O1—C1—H1A	109.5	C15—C16—H16A	109.5
C2—C1—H1A	109.5	C15—C16—H16B	109.5
O1—C1—H1B	109.5	H16A—C16—H16B	109.5
C2—C1—H1B	109.5	C15—C16—H16C	109.5
H1A—C1—H1B	108.0	H16A—C16—H16C	109.5
O2—C2—C1	113.6 (3)	H16B—C16—H16C	109.5
O2—C2—H2A	108.8	O4—C17—C18	124.6 (4)
C1—C2—H2A	108.8	O4—C17—C22	116.4 (3)
O2—C2—H2B	108.8	C18—C17—C22	118.9 (4)
C1—C2—H2B	108.8	C17—C18—C19	118.4 (4)
H2A—C2—H2B	107.7	C17—C18—C15	122.0 (4)
N1—C3—C6	121.0 (4)	C19—C18—C15	119.6 (4)
N1—C3—C4	119.0 (4)	C20—C19—C18	123.4 (4)
C6—C3—C4	120.0 (4)	C20—C19—H19	118.3
C3—C4—H4A	109.5	C18—C19—H19	118.3
C3—C4—H4B	109.5	C19—C20—C21	120.0 (4)
H4A—C4—H4B	109.5	C19—C20—H20	120.0
C3—C4—H4C	109.5	C21—C20—H20	120.0
H4A—C4—H4C	109.5	C26—C21—C22	118.7 (4)
H4B—C4—H4C	109.5	C26—C21—C20	122.2 (4)
O3—C5—C6	124.9 (4)	C22—C21—C20	119.1 (4)
O3—C5—C10	116.2 (4)	C23—C22—C21	118.8 (4)
C6—C5—C10	119.0 (4)	C23—C22—C17	121.0 (4)
C5—C6—C7	118.0 (4)	C21—C22—C17	120.1 (4)
C5—C6—C3	122.1 (4)	C24—C23—C22	121.2 (4)
C7—C6—C3	119.8 (4)	C24—C23—H23	119.4
C8—C7—C6	122.6 (4)	C22—C23—H23	119.4
C8—C7—H7	118.7	C23—C24—C25	119.7 (4)
C6—C7—H7	118.7	C23—C24—H24	120.1
C7—C8—C9	121.2 (4)	C25—C24—H24	120.1
C7—C8—H8	119.4	C26—C25—C24	120.3 (4)
C9—C8—H8	119.4	C26—C25—H25	119.9
C8—C9—C10	118.5 (4)	C24—C25—H25	119.9
C8—C9—C14	123.5 (5)	C25—C26—C21	121.3 (4)
C10—C9—C14	118.0 (5)	C25—C26—H26	119.3
C11—C10—C9	119.4 (4)	C21—C26—H26	119.3
			
O3—Cu1—N1—C3	−25.1 (4)	C14—C9—C10—C5	176.7 (4)
O4—Cu1—N1—C3	−106.3 (5)	O3—C5—C10—C11	2.8 (6)
N2—Cu1—N1—C3	138.7 (3)	C6—C5—C10—C11	−176.6 (4)
O3—Cu1—N1—O1	156.5 (3)	O3—C5—C10—C9	−175.8 (4)
O4—Cu1—N1—O1	75.2 (5)	C6—C5—C10—C9	4.7 (6)
N2—Cu1—N1—O1	−39.8 (3)	C9—C10—C11—C12	0.7 (6)
O3—Cu1—N2—C15	−51.8 (6)	C5—C10—C11—C12	−178.0 (4)
O4—Cu1—N2—C15	25.6 (4)	C10—C11—C12—C13	0.5 (7)
N1—Cu1—N2—C15	−172.7 (4)	C11—C12—C13—C14	−0.3 (8)
O3—Cu1—N2—O2	115.2 (4)	C12—C13—C14—C9	−1.1 (8)
O4—Cu1—N2—O2	−167.5 (3)	C8—C9—C14—C13	−179.1 (5)
N1—Cu1—N2—O2	−5.8 (3)	C10—C9—C14—C13	2.2 (7)
C3—N1—O1—C1	169.7 (4)	O2—N2—C15—C18	−174.5 (3)
Cu1—N1—O1—C1	−11.6 (4)	Cu1—N2—C15—C18	−7.2 (6)
C15—N2—O2—C2	−111.8 (4)	O2—N2—C15—C16	5.5 (5)
Cu1—N2—O2—C2	79.2 (3)	Cu1—N2—C15—C16	172.9 (3)
O4—Cu1—O3—C5	−166.2 (3)	Cu1—O4—C17—C18	30.4 (5)
N2—Cu1—O3—C5	−88.8 (5)	Cu1—O4—C17—C22	−151.3 (3)
N1—Cu1—O3—C5	33.8 (3)	O4—C17—C18—C19	179.4 (4)
O3—Cu1—O4—C17	125.2 (3)	C22—C17—C18—C19	1.2 (6)
N2—Cu1—O4—C17	−36.2 (3)	O4—C17—C18—C15	0.4 (6)
N1—Cu1—O4—C17	−153.3 (4)	C22—C17—C18—C15	−177.8 (3)
N1—O1—C1—C2	93.8 (4)	N2—C15—C18—C17	−12.2 (6)
N2—O2—C2—C1	−57.3 (5)	C16—C15—C18—C17	167.8 (4)
O1—C1—C2—O2	−55.4 (5)	N2—C15—C18—C19	168.8 (4)
O1—N1—C3—C6	−175.4 (3)	C16—C15—C18—C19	−11.2 (6)
Cu1—N1—C3—C6	6.0 (6)	C17—C18—C19—C20	−0.9 (6)
O1—N1—C3—C4	2.4 (5)	C15—C18—C19—C20	178.1 (4)
Cu1—N1—C3—C4	−176.1 (3)	C18—C19—C20—C21	0.5 (7)
Cu1—O3—C5—C6	−24.8 (6)	C19—C20—C21—C26	178.9 (4)
Cu1—O3—C5—C10	155.8 (3)	C19—C20—C21—C22	−0.4 (6)
O3—C5—C6—C7	176.0 (4)	C26—C21—C22—C23	−0.8 (6)
C10—C5—C6—C7	−4.6 (6)	C20—C21—C22—C23	178.5 (4)
O3—C5—C6—C3	−6.4 (6)	C26—C21—C22—C17	−178.6 (4)
C10—C5—C6—C3	173.0 (4)	C20—C21—C22—C17	0.7 (6)
N1—C3—C6—C5	15.5 (6)	O4—C17—C22—C23	2.8 (6)
C4—C3—C6—C5	−162.4 (4)	C18—C17—C22—C23	−178.9 (4)
N1—C3—C6—C7	−167.0 (4)	O4—C17—C22—C21	−179.5 (4)
C4—C3—C6—C7	15.2 (6)	C18—C17—C22—C21	−1.1 (6)
C5—C6—C7—C8	2.0 (6)	C21—C22—C23—C24	0.7 (6)
C3—C6—C7—C8	−175.6 (4)	C17—C22—C23—C24	178.5 (4)
C6—C7—C8—C9	0.6 (7)	C22—C23—C24—C25	0.3 (7)
C7—C8—C9—C10	−0.6 (6)	C23—C24—C25—C26	−1.4 (7)
C7—C8—C9—C14	−179.3 (4)	C24—C25—C26—C21	1.4 (8)
C8—C9—C10—C11	179.2 (4)	C22—C21—C26—C25	−0.3 (7)
C14—C9—C10—C11	−2.0 (6)	C20—C21—C26—C25	−179.5 (5)
C8—C9—C10—C5	−2.1 (6)		

### Hydrogen-bond geometry (Å, °)

**Table:**

*D*—H···*A*	*D*—H	H···*A*	*D*···*A*	*D*—H···*A*
C16—H16A···O3^i^	0.96	2.64	3.375 (5)	134
C23—H23···O2^ii^	0.93	2.43	3.261 (5)	149
C4—H4C···Cg8^i^	0.96	2.68	3.564 (6)	153

Symmetry codes: (i) −*x*+3/2, *y*+1/2, −*z*+3/2; (ii) −*x*+3/2, *y*−1/2, −*z*+3/2.
